# Determinants of US University Students’ Willingness to Include Whole Grain Pasta in Their Diet

**DOI:** 10.3390/ijerph18063173

**Published:** 2021-03-19

**Authors:** Rungsaran Wongprawmas, Giovanni Sogari, Davide Menozzi, Nicoletta Pellegrini, Michele Lefebvre, Miguel I. Gómez, Cristina Mora

**Affiliations:** 1 Department of Food and Drug, University of Parma, Parco Area delle Scienze 47/A, 43124 Parma, Italy; rungsaran.wongprawmas@unipr.it (R.W.); davide.menozzi@unipr.it (D.M.); cristina.mora@unipr.it (C.M.); 2 Department of Agricultural, Food, Environmental and Animal Sciences, University of Udine, Via Sondrio 9 2/A, 33100 Udine, Italy; nicoletta.pellegrini@uniud.it; 3 White Lodging School of Hospitality & Tourism Management, Purdue University Northwest, Hammond, IN 46323, USA; mlefebvre@pnw.edu; 4 Charles H. Dyson School of Applied Economics and Management, Cornell University, Ithaca, NY 14850, USA; mig7@cornell.edu

**Keywords:** whole grain pasta, college students, logistic regression, healthy eating

## Abstract

College students’ lifestyle and eating habits strongly affect their health. Among many healthy eating behaviors, including whole grain food in the diet is known as providing health benefits such as maintaining a steady blood sugar, lower cholesterol, and since it is rich in fiber and minerals, it is essential for the well-being. However, consumers’ intakes of whole grain products remain below recommendation, including college students. This study aims to evaluate determinant factors contributing to college students’ willingness to include whole grain pasta in their diets. A sample of 499 students enrolled in a US college participated in this study. Most students perceived whole grain pasta as healthy and filling and somewhat tasty. Availability and price were not barriers for consumption. Logistic regression results suggested that factors affecting students’ willingness to consume whole grain pasta in the future were the desire to eat, cognitive and affective attitudes, perception of whole grain pasta, as well as having already chosen pasta thanks to its availability at the dining. Two student segments were identified according to their healthy eating perception: Uninvolved and Health-conscious. Cognitive attitudes such as beneficial and essential had positive effects on consumption in both segments, suggesting that information provision covering specific health/nutritional benefits of whole grains for students is crucial.

## 1. Introduction

There are substantial socio-environmental changes when a young adult leaves home for the first time to attend college; this can be challenging for many reasons [1]. Moving to college requires young adults to start making their own food decisions for the first time in their lives. As such, this transition is often associated with unhealthy eating habits [2,3,4], which can contribute to the risk of overweight and obesity, and the associated diet-related diseases [5,6]. Indeed in the first years of college, freshmen and sophomore students in the United States have shown the tendency to gain weight due to sedentary lifestyles and excess calorie intake [6], and this may increase health risks if this trend continues throughout adulthood. These young adults are now faced with new dining settings, offering all you care to eat meals with an imbalance of healthy and unhealthy menu and food choices [7]. As such, students may consume less food considered to be healthy by the U.S. Department of Agriculture (USDA) Dietary Guidelines such as fruit, vegetables, and other high sources of dietary fiber such as whole grains (WG) (according to the U.S. Department of Health and Human Services and U.S. Department of Agriculture [8], Whole grains are grains and grain products made from the entire grain seed, usually called the kernel, which consists of the bran, germ, and endosperm. If the kernel has been cracked, crushed, or flaked, it must retain the same relative proportions of bran, germ, and endosperm as the original grain in order to be called whole grain. Many, but not all, whole grains are also sources of dietary fiber) and legumes [9].

Whole grains could help reduce the health risks because they are rich in fiber which could contribute to increasing the satiety sensations [10]. Feeling less hunger may help with weight loss or prevent weight gain. In addition, whole grains are also rich in nutrients such vitamins, minerals, and antioxidants [11] which contribute to the reduction of tiredness and fatigue [12]. Evidence has shown how high intakes of whole grain and dietary fiber are linked to a low incidence of several non-communicable diseases, including obesity and overweight-related diseases [13,14], certain cancers, type 2 diabetes, and cardiovascular diseases [15].

For such and other health benefits (see Jones and Engleson, 2010 for a more comprehensive review), governmental institutions and nutritional experts have developed nutrition education and health promotion campaigns to recommend including whole grain in the diet [16,17,18]. For instance, the 2020–2025 Dietary Guidelines for Americans suggests that a healthy diet should include grains, at least half of them from whole grains [9].

Although recent data [19] showed that the consumption of whole grains to total grains intake increased for adults from 12.6% in 2005 to 15.9% in 2016, the intake recommended by US dietary guidance (48 g/day or three servings) is still not reached from most of the general population [20]. Even young adults, e.g., college students, include low amounts of whole grain in their diet [21]. Rose et al. (2007) indicated that among American college students, whole grain intake was less than one daily serving [22].

In order to increase college students’ whole grain consumption, the “Menus of Change” program was introduced in 2012. This program focuses on achieving healthy and sustainable menus, with the tagline of “The Business of Healthy, Sustainable, and Delicious Food Choices”. This program was founded by the Culinary Institute of America and was meant to promote health and sustainability within any foodservice operation type. Shortly after this initiative was launched, a campus focused program was created, entitled the Menus of Change University Research Collaborative (MCURC); this was funded jointly by Stanford University and The Culinary Institute of America [23]. This program strives to promote health and sustainability within any foodservice operation type and wrote guidelines focusing on achieving healthy and sustainable menus. As such, the primary goal for the MCURC is to use campus dining venues as a platform for education and learning around the Menus of Change principles and thus to educate students and prepare them for a lifetime of healthy and sustainable food decisions. Some of the core principles are in line with the USDA’s 2020–2025 Dietary Guidelines and include making whole, intact grains the new norm, and focusing on whole, minimally processed foods. Both of these guidelines promote the inclusion of more whole grains on the menu.

Several barriers to increasing whole grain food consumption have been identified in the past such as limited market availability and rejection to taste and texture [24,25,26]. Therefore, in the past decade public health and government measures (e.g., ‘U.S. dietary guidance’ or the ‘Health Promotion and Disease Prevention’ guidelines from the EU) have tried to promote whole grain foods through the development of healthy nutritional profiles [16,27,28,29]. According to the “Global Consumers Trends” report [30], this promotion has increased the consumers interest for high-fiber foods such as whole grains [16,31,32]. Therefore, manufacturers increased the supply of whole-grain foods with new or reformulated products to meet consumer’s needs [27,28].

However, limited research has been given to consumer attitudes and perceptions towards whole grains, especially among young university students. Attitudes are very important to determine food choices. For instance, the negative perception of whole grain sensory attributes (i.e., taste and texture) have been identified as a barrier to consume such products [33].

One food product made with wholegrains that can demonstrate increased acceptability in meeting recommended whole grain intake is pasta. Pasta is a key component of the Mediterranean Diet, and its consumption has been positively associated with a low body mass index and prevention of overweight and obesity risk conditions [34,35,36]. Pasta is a staple food in Mediterranean countries and it became very popular all over the world [37]. One hundred grams of uncooked whole grain pasta contains around 6–10 g of fiber [38,39,40].

Previous studies [21,41] showed that nutrition point-of-selection messaging in campus dining halls is an effective strategy to increase whole grain intake among US college students. However, sometimes intervention studies provide little insight regarding the behavioral reasons for choosing whole grain vs. regular products. In order to better establish tailored intervention recommendations to increase whole grain consumption, it is important to understand the factors that drive consumers toward this choice [42]. As such, a study exploring individual factors that could impact the intention to choose or purchase whole grain pasta among university students is needed, as their consumption habits now could shape their habits in the future.

The purpose of this study was to evaluate determinant factors contributing to university students’ willingness to include whole grain pasta in their diet. More specifically, it aims to identify (1) different groups of students according to their healthy eating perception, and (2) the main factors associated to the consumer perception of whole grain pasta, in each group, based on the barriers/facilitators for whole grain consumption, such as cost differences, lack of availability, sensory appeal [33,42] and others, such the perceptions of feeling fuller and being healthier [43]. Finally, the study aims to evaluate the role of attitudes toward including whole grain pasta in the diet, such as benefits and importance [43].

## 2. Materials and Methods

### 2.1. Data Collection

This study was conducted in collaboration with the Cornell Dining Center which provided access to the university dining venues where the data collection took place. The study was approved by the Institutional Review Board (IRB) of the Office of Research Integrity and Assurance of Cornell University (Protocol Number: 1810008359). The entire study was conducted between February and April 2019, during dinner hours (from 5 pm to 8 pm) in two dining venues (Robert Purcell Marketplace Eatery and North Star Dining Room). Data were collected using Qualtrics online survey software (Qualtrics, LLC; Seattle WA). To ensure that data collection procedures in the field were consistent, research assistants were specifically trained and instructed on how to collect questionnaires.

Only individuals who chose pasta as main meal were asked to participate to the study (i.e., pasta consumers) and instructed on the study procedures by two investigators. First, they were told the study goals (i.e., find out more about the relationships between student’s eating behavior and pasta consumption) and were told that they could remove themselves from the study at any time without any disadvantage. Second, they were asked to give their consent for participation and were informed that data confidentiality was assured.

After completing a short preliminary questionnaire (Section 1—Preliminary questions), they were told to eat dinner as usual and that, after eating, they would have received an email with the main survey to be filled out. Following the completion of the study, participants received a monetary compensation of USD 5.

There were originally 514 students who completed the preliminary questionnaire. Fifteen participants were excluded because they took too little time to complete the survey (i.e., less than the one third of the average duration) or they did not complete the questionnaire. The final sample consisted of 499 individuals.

### 2.2. Questionnaire Content

The initial questionnaire was based on a review of the existing literature followed by a revision of two experts in nutrition and diet and three experts in social sciences. The study was also guided by previous focus groups on healthy diet and eating habits with university students [2]. The entire survey was pre-tested before launching it with 10 students and 5 Faculty staff members of Cornell University. Several questions were revised to improve the clarity of their meaning and to reduce the total survey length to approximately a 12-min duration.

The questionnaire consisted of five sections: Section 1—Preliminary questions; Section 2—Attitudes related to healthy eating; Section 3—Pasta and wholegrain consumption habits; Section 4—Perceptions of whole grain pasta, attitude toward and willingness to include whole grain pasta in the diet; and Section 5—Socio-demographical data. The questionnaire comprised both closed-ended and opened-ended questions. In most questions, participants were asked to give their responses according to a 7-point semantic scale. The individual items and the type of scale for all measures are provided in Appendix A (Table A1).

In the first section, before eating, participants were asked to indicate their level of hunger, fullness, desire to eat and expected pleasant levels, ranging from 1 (not at all) to 7 (extremely). They were then asked to indicate actual pasta choice at dinner.

In the second section, attitudes related to healthy eating consisted of “self-reported healthy eating” (adapted from Van Loo et al., 2017 [44]), “interest in healthy eating” (adapted from Hung et al., 2010 [45]) and “health concern” (adapted from Pieniak et al., 2010 [46]). Participants were asked to indicate to what extent the statements applied to them, ranging from 1 (Does not apply to me at all) to 7 (Fully applies to me).

In the third section, to measure the consumption of pasta, participants were asked to indicate, on average, how frequently they consumed regular (regular pasta is made from refined flours, such as wheat flour. The milling process involves stripping the grain of its bran and germ, which gives the flour a finer texture, but also alters the nutritional content of the grain) and whole grain pasta (whole-grain pasta is made from whole-grain flour where most of the bran and germ are retained in the pasta), ranging from 1 (never) to 7 (every day). Participants were also asked to indicate the importance of pasta attributes (type of cut, type of topping, wholegrain and healthiness) when choosing pasta, ranging from 1 (not at all important) to 7 (extremely important).

In the fourth section, six 7-point semantic differential items were used to measure perception in response to the following statement: ‘In your opinion, a whole grain pasta is…’. The six items used the following endpoints: not tasty/tasty, cheap/expensive, not easily available/easily available in the store I usually shop, not filling/filling, not healthy/healthy, not easily available/easily available in the dining hall I usually eat (adapted from Van Loo et al. 2017 [44]). Attitudes toward the inclusion of whole grain pasta in the diet were measured based on the likelihood that consuming whole grain pasta would result in specific personal beliefs (i.e., beneficial, wise, essential, easy and tasty). Five 7-point semantic differential items were used to measure attitudes in response to the following statement: ‘Including whole grain pasta in my diet over the next month will be…’. The five items used the following endpoints: harmful/beneficial, foolish/wise, unnecessary/essential, difficult/easy, and not tasty/tasty (adapted from Menozzi et al. 2015 [47] and Jun et al. 2016 [48]). Participants were also asked to indicate how likely would they be to willingly start including whole grain pasta in the diet over the next month, ranging from 1 (extremely unlikely) to 7 (extremely likely).

The final section included socio-demographic data, such as participants’ age, gender, country of origin, and ethnic/racial identity. Other variables assessed were self-reported physical activity (i.e., duration in a week) and weight (pounds) and height (feet and inches). Finally, to assess individual relationship with food in general, a series of dietary questions were posed (e.g., dietary/health restrictions, allergies, specific dietary regimen like veganism or vegetarianism).

### 2.3. Data Analysis

Descriptive statistics were used to report percentages, medians, means and standard deviations of variables collected through the survey. Body mass index (BMI) was calculated using self-reported converted height (m) and weight (kg) data. The results of the BMI were classified according to International Classification Standards [49]: underweight (BMI < 18.50 kg/m^2^), normal weight (18.50 ≤ BMI ≤ 24.99 kg/m^2^), overweight (25.00 ≤ BMI ≤ 29.99 kg/m^2^) and obese (BMI ≥ 30.00 kg/m^2^). Missing values of BMI were replaced by average BMI of the sample.

Consumer groups were identified using the data contained in Section 2—Attitudes relating to healthy eating. Principal components analysis (PCA) with varimax rotation was performed separately on self-reported healthy eating (HE), interest in healthy eating (IHE), and health concern (HC). The results are shown in Appendix A (Table A2). Cronbach’s alpha was used to assess the internal reliability and consistency of the multi-item scales. The factors, then, were used in the cluster analysis. Hierarchical cluster analysis (HCA), with squared Euclidean distances, Ward’s method was applied to the items in order to define the optimum number of clusters. Then, K-means cluster analysis was used to identify final cluster results. Segments were profiled by comparing their socio-demographic, anthropometrics, health related behaviors, consumption frequency of pasta, importance of pasta attributes, including attitude and perception of wholegrain pasta. For the comparison between clusters, the Student’s t-tests and Mann–Whitney U tests were used.

A logit regression was used to examine the factors influencing participants’ willingness to include whole grain pasta in the diet. The dependent variable was the responses of the question, ‘how likely would you be willing start including whole grain pasta in your diet over the next month’. The responses of the question were recoded from 7-semantic scale to binary responses, 0 = no (1 = extremely unlikely to 4 = neither likely nor unlikely) and 1 = yes (5 = slightly likely to 7 = extremely likely), respectively. The explanatory variables comprise actual pasta choice at dinner, desire to eat, BMI category, cognitive attitude, affective attitude, and perception of whole grain pasta. Actual pasta choice at dinner was a categorical variable (1 = regular; 2 = wholegrain; 3 = other types of pasta). Desire to eat which was a categorical variable was recoded to a dummy variable, 0 = no (1 = very weak to 4 = neutral) and 1 = yes (5 = moderately strong to 7 = very strong). BMI category was a categorical variable (1 = underweight, 2 = normal weight, 3 = overweight, 4 = obese). A factor analysis was performed on attitude and perception of wholegrain pasta variables and the results are shown in Appendix A (Table A3). Therefore, cognitive attitude, affective attitude and perception of whole grain pasta were continuous variables. Other demographics, habits, and opinion variables had been also introduced in the models, to simultaneously control for their influence on willingness to include whole grain pasta in the diet. However, due to lack of significance and poor fit, they were not included in the final models.

## 3. Results

### 3.1. Sample Characteristics

#### 3.1.1. Socio-Demographic and Anthropometric Data

Descriptive statistics for the pooled sample are presented in Table 1. All participants were enrolled at the university, the mean age was 18.8 ± 1.2 years old, 53.7% female, a representative mix of ethnicities for the geographic area (50.9% White/Caucasian, 26.9% Asian excluding South Asian, 12.8% Hispanic/Latino, 11.4% Black/African American, 9% South Asian, and 8.4% in other categories). Most of the participants’ country of origin was the US (86.4%), following by China (4.0%), India (1.4%) and other countries (8.2%). Samples were representative of the Cornell University’s undergraduate students (Consensus Fall 2020, [50]) in terms of gender, age and ethnicities.

Participants had an average BMI, calculated based on self-reported height and weight, of 22.9 ± 5.7. BMI was normal ranged for 49.7% of participants, while 18.4% were overweight, 9.3% were obese and 22.4% were underweight. Most participants (62.9%) reported highly active, practicing physical activity for at least 2–3 times per week, while 34.7% were categorized into inactive (never to once a week). The majority of participants (80.0%) did not follow any food regimen, while 9.0% was flexitarian, 6.6% vegetarian, 3.2% vegan, 0.6% raw foodie and 0.6% fruitarian. Most of the participants (83.8%) did not have any food allergies/intolerances. Most participants reported self-perception of overall health as moderately well. Differences between the two clusters (Uninvolved and Health Conscious) are discussed below after the cluster analysis results are presented.

#### 3.1.2. Pasta Consumption Habits

Table 2 presents descriptive statistical results of pasta consumption habits. On average the participants reported that they consumed regular pasta once a week (median = 5) while they consumed whole grain pasta 2–3 times per month (median = 4). Regarding importance of pasta attributes, type of topping was reported as very important (median = 6), followed by type of cut (i.e., shape of pasta) (median = 5, moderately important), wholegrain and healthiness (median class = 4, neutral), respectively. Most of participants indicated that they were slightly likely to willingly start including whole grain pasta in their diet over the next month (median = 5).

The majority of participants had actually chosen regular pasta (54.5%), following by whole grain pasta (42.1%) and other types of pasta (e.g., egg pasta, stuffed pasta) (3.4%) at dinner before filling the questionnaire.

#### 3.1.3. Perception of Whole Grain Pasta and Attitude of Including Whole Grain Pasta in the Diet

**Figure 1:**
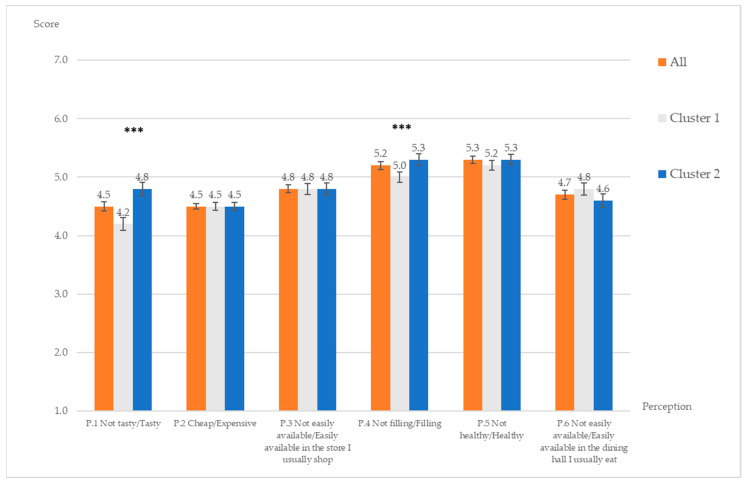
Perception of whole grain pasta in the sample, Cluster 1 (Uninvolved) and Cluster 2 (Health-conscious). Results from Mann–Whitney U test between 2 clusters *** significant at the 0.01 level.

**Figure 2:**
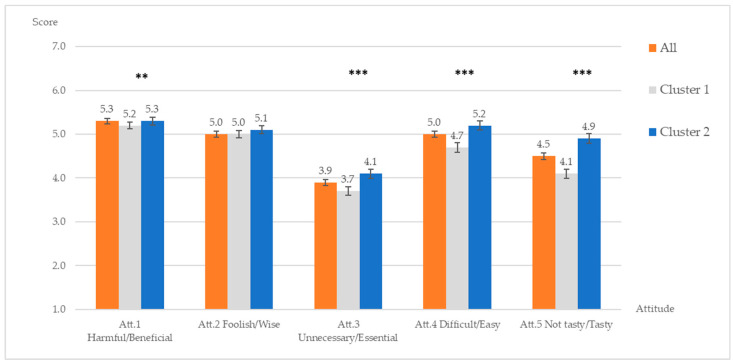
Attitude towards including whole grain pasta in the diet over the next month in the sample, Cluster 1 (Uninvolved) and Cluster 2 (health-conscious). Results from the Mann–Whitney U test between 2 clusters: ** significant at the 0.05 level, *** significant at the 0.01 level.

Figure 1 and Figure 2 present results of perception of, and attitude towards whole grain pasta, respectively. Participants perceived that whole grain pasta is somewhat tasty, filling and healthy, while they neither agree nor disagree that it is expensive. Participants also reported that whole grain pasta could be available both in the store they usually shop and in the dining hall they usually eat. Participants believed that including whole grain pasta in their diet over the next month would be somewhat beneficial, somewhat wise and somewhat easy, while they were neutral about essential and taste aspects of it.

### 3.2. Segmentation of Consumers

Consumer groups were identified starting from the participants’ perception of healthy eating. First, an exploratory factor analysis (principal components, varimax rotation) was performed separately on three questionnaire parts defining respondents’ perception of healthy eating, i.e., self-reported healthy eating, interest in healthy eating, and health concern. The results of the factor analysis are shown in Appendix A (Table A2). Overall, 12 questionnaire items were retained. Seven items loaded on the “Healthy Eating” (HE) factor, which explained 43.9% of the variance (HE.2 “I eat bread, grains, pasta, rice or potatoes several times per day” was excluded from the final factor analysis, see details in Appendix A). This factor consisted of statements regarding the consumers’ healthy eating behaviors, such as eating a variety of fruit and vegetables, replacing meat products with alternative proteins (e.g., legumes), controlling the salt intake, etc. Two items were used to assess consumers’ “Interest in healthy eating” (IHE). This factor explained 81.3% of the total variance of two statements regarding respondents’ perception of following a healthy and balanced diet, and their interest in the healthiness of food. Finally, the third factor, “Health concern” (HC), was able to explain 79.1% of the variance of three items regarding consumers’ concerns in their own health. These three factors plus the single reversed item “Care about healthiness” (standardized of reversed IHE.3) were entered into a cluster analysis.

A K-mean cluster analysis was applied on the four factors to identified final clusters (Table 3). The two clusters of consumers were named as Uninvolved and Health-conscious.

The first cluster (*n* = 233, 47%) consisted of consumers that are consistently Uninvolved and less interested in healthy-eating, and that are in general less concerned about their health. As shown in Table 1 they are less likely to be white and have more often a normal food regime, instead of being vegetarians or flexitarians. Regarding pasta consumption, as shown in Table 2, these Uninvolved consumers are more often eating regular than wholegrain pasta, are indifferent about including wholegrain pasta in their diet, and are less interested in pasta attributes such as wholegrain, type of topping, and healthiness than Health-conscious consumers. The cluster with Health-conscious consumers (*n* = 266, 53%) encompassed participants that are more aware of the consequences of healthy eating, are more likely carrying out healthy eating behaviors, and are generally more concerned about healthiness. These consumers are more often white, involved in some dietary regime (e.g., vegetarian or flexitarian), and perceived to be healthier than the average (Table 1). Table 2 shows that these consumers are more often eating wholegrain pasta and are willing to include it in their diet than Uninvolved consumers. Moreover, besides their interest in the wholegrain attribute, Health-conscious consumers are more often searching for other pasta characteristics such as type of topping, and healthiness than Uninvolved counterparts.

The overall more positive perception and attitude towards wholegrain pasta consumption of the Health-conscious segment compared to the Uninvolved cluster is driven by beliefs that wholegrain pasta is more beneficial, essential, easy to handle, tasty and filling (Figure 1 and Figure 2). Not surprisingly, a large share of participants in the Health-conscious cluster have actually chosen wholegrain pasta at dinner before filling the questionnaire compared to those in the Uninvolved segment (51.5% vs. 31.3%, respectively), whereas the opposite is true for regular pasta (65.7% vs. 44.7%, respectively) (z-test = −4.537, *p* < 0.001).

### 3.3. Willingness to Include Whole Grain Pasta in the Diet

Table 4 reports the results of the logit regression aiming at exploring the factors influencing participants’ willingness to include whole grain pasta in the diet. The dependent variable is the response of the question, ‘how likely would you be willing start including whole grain pasta in your diet over the next month’, recoded to binary variable. The explanatory variables are Actual pasta choice, Desire to eat, BMI category, Cognitive attitude, Affective attitude, and Perception of whole grain pasta. The three last variables were derived from factor analyses (PCA, varimax rotation), the results of which are shown in Appendix A (Table A3). Cognitive attitude includes items related with the effects of eating wholegrain pasta, while affective attitude is related with person’s feelings such as taste and easiness.

The results indicate that the willingness to start including whole grain pasta in the diet for students in the Uninvolved cluster is strongly positively affected by cognitive attitude (*p* < 0.001), and affective attitude (*p* = 0.001). Their willingness to include whole grain pasta in the diet is also positively affected by perception of whole grain pasta (*p* < 0.01) and, to a lesser extent, by the desire to eat pasta (*p* < 0.05). This means that the willingness to include whole grain pasta in the diet for those uninvolved with healthy eating is stronger, the more positive is the attitude, both cognitive and affective, and the perception of whole grain pasta, and the higher is the desire to eat pasta.

The willingness to include whole grain pasta in the diet for the Health-conscious cluster is positively related with the actual wholegrain over regular pasta choice (*p* < 0.001). In other words, Health-conscious consumers that are already choosing wholegrain pasta are more willing to include it in their diet. Moreover, cognitive attitude (*p* < 0.01), being overweight instead of normal weight (*p* < 0.05) and having a stronger desire to eat pasta (*p* < 0.05) are also positively influencing the participant’s willingness to include whole grain pasta in their diet.

## 4. Discussion

Participants perceived that whole grain pasta as healthy and filling and somewhat tasty in accordance with other studies [42,51,52]. Availability of whole grain pasta and price which were the main barriers of whole grain consumption in previous studies [51,52,53,54,55] were not important issues for the participants since they could find whole grain pasta both at shopping places and dining halls. This might be because at the dining venues of Cornell University, whole grain pasta is usually available at the same price of regular pasta, thanks to the Eating Well with Cornell Dining program [56]. The Eating Well with Cornell Dining program was founded in 2010 and the aim was to include more healthy menu options including whole grain products across all dining locations to promote healthier diets among college students. Since one of the requirements of the program was for the menu item to be 100% whole grain, whole grain pasta soon became commonplace across menus on the campus alongside regular pasta options. This confirms that if whole grain pasta is available with competitive price (or equal to the price of regular pasta), the consumption of whole grain pasta would increase [42,57], as around 40% of study participants actually chose whole grain pasta for dinner.

Two distinct consumer segments were identified based on differences in involvement in healthy eating. More than half of the participants were Health-conscious and about 47% of the participants were Uninvolved. Participants belonging to the Health-conscious segment are more health concerned, interested in healthy eating and following a healthy eating scheme. As expected, a higher proportion of participants in the Health-conscious segment chose whole grain pasta at dinner and were more willing to include whole grain pasta in the diet than participants in the Uninvolved segment. While healthiness of pasta is an important attribute (median = 5) for the Health-conscious participants, wholegrain attribute is somewhat important (median = 4) for them. However, the type of topping was the most important attributes for both segments even though there was a significant difference between the clusters. The Health-conscious segment’s average score for this attribute is significantly higher than those of the Uninvolved segment. This is consistent with Naessens (2018), who suggested that whole grain pasta dishes can be part of a healthy eating pattern as long as it is consumed in a moderate portion with the healthy types of sauces and topping, e.g., adding plenty of vegetables and/or unprocessed meats or plant based protein sources such as tofu or beans [58].

Regarding perception of whole grain pasta, the Uninvolved participants were uncertain that whole grain pasta is tasty or filling while the Health-conscious participants scored significantly higher in these aspects. This is consistent with the results of Magaris et al. (2016) which conducted a survey of college students and found the students grouped in medium and high whole grain intake categories scored higher for sensory liking of whole grain pasta compared to those with low whole grain intake [54]. Bisanz and Krogstrand (2007) confirmed that taste was a main barrier of whole grain food consumption in the college student [51].

When study participants were asked about attitudes toward including whole grain pasta in the diet in the next month, they thought it would be beneficial, wise, and easy to do. However, they neither agreed nor disagreed that it would be tasty. Differences between the two segments were that Health-conscious participants reported significantly higher scores on beneficial, easy, tasty and essential of including whole grain pasta in the diet than Uninvolved ones. Nevertheless, both segments were uncertain that it would be necessary (essential) to include whole grain pasta in the diet. This confirms results from previous studies [54,59,60] that awareness of benefits of consuming whole grain food will not necessary lead to college students’ decision to include it in their diet.

To better understand this issue, the analysis of factors affecting willingness to include whole grain pasta was examined in this study. Factors that contribute to participants’ willingness to include whole grain pasta were the desire to eat, cognitive and affective attitude, the perception of whole grain pasta, as well as having already chosen pasta thanks to its availability at the dining. In other words, subjects who actually consumed whole grain pasta were more likely to add whole grain pasta to their diet in the next month, confirming the findings of similar studies [54]. Participants who reported high desire to eat were also more likely to choose whole grain pasta. Attitudes also have impact on whole grain consumption [54,55,59].

Although both affective and cognitive attitudes were significant predictors of willingness to include whole grain pasta, cognitive attitudes (i.e., beneficial, wise, essential) had a greater effect than affective attitudes (i.e., easy, tasty). This implied that if the participants receive more rational information about benefits and essentials of whole grain pasta, they will be more likely to include whole grain pasta in the diet. This finding is in contrast with the result of Jun and Arendt (2016) who found that affective attitudes had a stronger effect than cognitive attitudes on behavioral intentions and willingness to choose healthful menu items [48]. However, Trendel and Werle, 2016 conducted studies about the effect of affective and cognitive attitudes on food choice and found that both types of attitude influenced food choices under different circumstances [61]. On the one hand, when cognitive information (e.g., nutritional facts) is limited, affective basis of implicit attitudes is the only driver of food choice. On the other hand, when cognitive information is available, the cognitive basis influences food choice but only for participants low in impulsivity [61]. Future research should measure how cognitive and affective attitudes affect the intention and actual behavior of whole grain consumption.

Note that for Health-conscious segment, only cognitive attitude seems to influence the willingness to include pasta in the diet, whereas affective attitude and perception of whole grain pasta do not have any effect. The willingness to include whole grain pasta in the diet of the Health-conscious consumers, who are already more aware of the consequences of healthy eating and are more likely carrying out healthy eating behaviors, is not affected by the consumers’ perception of whole grain per se, nor by the expected taste or easiness of eating whole grain pasta. These factors are instead significant predictors of the stronger willingness to include whole grain pasta in the diet of Uninvolved consumers. In addition, findings demonstrated that, within the Health-conscious segment, being overweight increased the willingness to include whole grain pasta in the diet. This result is unexpected since a previous study [22] found that whole grain intake was significantly higher in normal weight students than in overweight and obese students. The possible explanation could be that Health-conscious participants know about the effect of fiber on weight; therefore, overweight participants may want to eat whole grain to lose weight.

The Uninvolved segment seems to be a more challenging group when it comes to promoting whole grain pasta consumption. They think that including whole grain pasta in the diet will not be that essential nor beneficial. In addition, this segment was not overly health-oriented and perceived whole grain pasta as not tasty or filling. However, this perception is common as several studies [42,60,62,63] show that adults have a pre-existing negative image of whole grain-containing products that could be improved with tasting and familiarization with them.

The Health-conscious segment is a promising group for promoting whole grain pasta consumption. The members in this group perceived whole grain pasta to be tasty and filling, and healthfulness was an important attribute when choosing pasta. Moreover, they reported themselves as health-conscious and perceived higher need to pay attention to the healthiness of their diet which provides them both reason and motivation to consume whole grain pasta.

It could be interesting to examine whole grain intakes among students from different countries. For example, future studies can collect data to compare the results of the U.S. with university students in Mediterranean countries, such as Italy, where pasta, not whole grain one, is a traditional staple food. Differences among countries would be likely influenced by habits, attitudes, messages, and nudging interventions.

Some limitations should be acknowledged when interpreting our findings, which identify some opportunities for further research. The interpretation of our results to general college students in the US should be done with care since in Cornell’s all you care to eat locations the whole grain pasta option is always available at the same price of regular pasta; this context might be different in other US colleges and in the other countries. By choice, we did not test the subject’s knowledge about wholegrain (ability to identify wholegrain products), and we did not ask about their awareness of recommended wholegrain quantity which are two main barriers from previous literature [42,52], hence these issues should be included in further research.

## 5. Conclusions

This study confirms that availability of whole grain option with comparable price to the conventional option could be beneficial for students since it could mitigate consumption barriers (availability and price issues) and promote whole grain pasta consumption in college dining halls. The choice of students for pasta could be influenced not only by whole wheat or regular types but also by the type of toppings. This should be taken into consideration when developing recipes’ concept.

Since cognitive attitudes are crucial in promoting whole grain pasta for both Health-conscious and Uninvolved segments, information provision covering specific health/nutritional benefits of whole grains for students is crucial [41]. Since affective attitudes also influenced Uninvolved segment, it means that individual’s feelings or emotions toward whole grain pasta appear to be critical in the decision to select them. Hence, the promotion campaigns should incorporate affective components to highlight pleasurable attributes (e.g., tasty) of whole grain pasta.

Considering that the sample is from one college, our results cannot be generalized to the US college population, but this study could be applied in other colleges and in different countries to further investigate this issue on a global scale.

## Figures and Tables

**Table 1:** Socio-demographic and anthropometric (%) characteristics of the total sample, Cluster 1 and Cluster 2.

Characteristics	Heading	All	Cluster 1 Uninvolved	Cluster 2 Health-Conscious	*p*-Value
	*n*	499	233	266	
	%		47	53	
Gender	Median	Female	Female	Female	0.567 ^b^
	Male	44.3	42.9	45.5	
	Female	53.7	54.9	52.6	
	Other	2.0	2.2	1.9	
Age	Mean (SD)	18.8 (1.16)	18.7 (0.97)	18.8 (1.30)	0.431 ^a^
Country of origin	Median	US	US	US	0.418 ^b^
	US	86.4	88.0	85.0	
	China	4.0	4.3	3.8	
	India	1.4	0.9	1.9	
	Canada	0.8	0.4	1.1	
	Nigeria	0.8	1.3	0.4	
	Others	6.6	5.1	7.8	
Ethnicity ^1^	White	50.9	45.9	55.3	0.037 ^b^
	Asian, excluding South Asian	26.9	25.8	27.8	0.603 ^b^
	Hispanic/Latino	12.8	11.6	13.9	0.439 ^b^
	Black or African American	11.4	13.7	9.4	0.129 ^b^
	South Asian	9.0	9.0	9.0	0.997 ^b^
	Others	8.4	7.3	5.6	-
BMI	Mean (SD)	22.9 (5.72)	23.2 (6.15)	22.7 (5.31)	0.385 ^a^
BMI Category	Median	Normal	Normal	Normal	0.390 ^c^
	Underweight (BMI < 18.50)	22.4	21.9	22.9	0.709 ^b^
	Normal weight (18.50 ≤ BMI ≤ 24.99)	49.7	48.5	50.8	0.705 ^b^
	Overweight (25.00 ≤ BMI ≤ 29.99)	18.4	18.0	18.8	0.705 ^b^
	Obese (BMI ≥ 30.00)	9.3	11.6	7.5	0.725 ^b^
Physical Activities	Median	High	High	High	0.667 ^c^
	Low (Never to once time per week)	34.7	33.9	35.3	
	High (At least 2–3 times per week)	62.9	64.8	61.3	
	Do not want to answer	2.4	1.3	3.4	
Food regimens	Median	Normal	Normal	Normal	0.001 ^b^
	Normal	80.0	87.6	73.3	
	Vegetarian	6.6	3.9	9.0	
	Vegan	3.2	0.4	5.6	
	Flexitarian	9.0	6.9	10.9	
	Raw Foodie	0.6	0.4	0.8	
	Fruitarian	0.6	0.9	0.4	
Allergies	Median	No	No	No	0.492 ^b^
	Yes	16.2	15.0	17.3	
	No	83.8	85.0	83.7	
Self-perception of overall health ^2^	Mean (SD) Median	4.4 (0.34) 5	4.4 (1.44) 5	5.5 (1.10) 6	<0.001 ^c^

^1^ Participants could choose multiple ethnic groups. ^2^ participants were asked to make self-report on how healthy they were based on 7-semantic scale (1 = very bad, 7 = very well). ^a^ Student T-test. ^b^ Pearson chi-square. ^c^ Mann–Whitney U Test.

**Table 2:** Consumption frequency of pasta and attributes that affect the pasta consumption of the total sample, Cluster 1 and Cluster 2.

Item	All			Cluster 1 Uninvolved			Cluster 2 Health-Conscious			*p*-Value ^4^
	Median	Mean	SD	Median	Mean	SD	Median	Mean	SD	
**Consumption frequency of pasta ^1^**										
Regular	5	4.7	1.33	5	4.9	1.31	5	4.6	1.34	0.007
Wholegrain	4	3.8	1.52	4	3.5	1.56	4	4.2	1.42	<0.001
**Pasta attribute ^2^**										
Type of cut	5	4.0	1.73	5	4.0	1.72	4	4.0	1.75	0.801
Wholegrain	4	3.7	1.66	3	3.2	1.59	4	4.2	1.59	<0.001
Type of topping	6	5.3	1.42	5	5.1	1.47	6	5.4	1.35	0.004
Healthiness	4	4.0	1.59	4	3.4	1.48	5	4.4	1.53	<0.001
**Willingness to include wholegrain pasta ^3^**	5	4.4	1.89	4	4.0	1.82	6	4.8	1.86	<0.001

Note: ^1^ The consumption frequency was measured on a 7-semantic scale (1 = never, 2 = less than 1 time/month, 3 = 1 time/month, 4 = 2–3 times/month, 5 = 1 time/week, 6 = several times/week, 7 = everyday). ^2^ The importance of each attribute was measured on a 7-semantic scale (1 = not at all important, 7 = extremely important). ^3^ The willingness to start including whole grain pasta in the diet over the next month was measured on a 7-semantic scale (1 = to extremely likely, 7 = extremely likely) ^4^ Mann–Whitney U Test between 2 clusters.

**Table 3:** Categories of final clusters in the sample.

Factor	Cluster	
	1 Uninvolved (*n* = 233)	2 Health-Conscious (*n* = 266)
**Healthy Eating**	−0.685	0.600
**Interest in Healthy Eating**	−0.774	0.678
**Care about healthiness**	−0.518	0.454
**Health Concern**	−0.730	0.639

**Table 4:** Factors determining willingness to include wholegrain pasta in the diet.

Variable	Cluster 1 (*n* = 233) Uninvolved			Cluster 2 (*n* = 266) Health-Conscious		
	Coef.	SE	*p*-Value	Coef.	SE	*p*-Value
Intercept	−0.921	0.365	0.012	−0.629	0.289	0.030
Actual pasta choice ^1^						
Wholegrain pasta	0.589	0.348	0.091	1.131	0.305	<0.001
Other pasta	1.012	0.989	0.306	0.423	0.732	0.564
Desire to eat	0.786	0.345	0.023	0.607	0.292	0.038
BMI category ^2^						
Underweight	−0.363	0.423	0.390	0.001	0.343	0.998
Overweight	0.227	0.431	0.599	0.982	0.428	0.022
Obese	0.148	0.499	0.767	0.781	0.595	0.189
Cognitive attitude	0.931	0.212	<0.001	0.447	0.141	0.002
Affective attitude	0.608	0.187	0.001	0.164	0.164	0.317
Perception of whole grain pasta	0.562	0.204	0.006	0.133	0.141	0.345
Number of participants	233			266		
Log likelihood	−124.1			−148.5		
McFadden’s pseudo R^2^	0.216			0.155		

Note: ^1^ baseline = regular pasta, ^2^ baseline = normal weight.

## Appendix A. Overview of Measures Used in the Study and Factor Analysis Results

**Table A1:** Overview of measures used in the study.

Measures	Item/Statement	Scale
**Preliminary questions**		
Desire to eat	How strong is your desire to eat pasta now?	7-point scale from “Very weak” (1) to “Very strong” (7)
Actual pasta choice	which kind of pasta did you eat for dinner today?	1 = regular pasta, 2 = whole grain pasta, 3 = other types of pasta
**Healthy Eating**	Please indicate to what extent the following statements generally apply to you.	7-point scale from “Does not apply to me at all” (1) to “Fully applies to me” (7)
HE.1	I eat a variety of foods originating mainly from plants, rather than animals	
HE.2	I eat bread, grains, pasta, rice or potatoes several times per day	
HE.3	I eat a variety of vegetables and fruits, at least 0.9 lb (400 g) per day or 5 portions per day	
HE.4	I control my fat intake and replace most saturated fats with unsaturated vegetable oils or soft margarines	
HE.5	I replace fatty meat and meat products with beans, legumes, lentils, fish, poultry or lean meat	
HE.6	I select foods that are low in sugar, limiting the frequency of intake of sugary drinks and sweets	
HE.7	I control my salt intake and limit adding salt to my meals	
**Interest in Healthy Eating**	Please indicate to what extent the following statements generally apply to you.	7-point scale from “Does not apply to me at all” (1) to “Fully applies to me” (7)
IHE.1	I always follow a healthy and balanced diet	
IHE.2	I am very particular about the healthiness of food	
IHE.3	I eat what I like and I do not worry about the healthiness of food	
**Health Concern**	Please indicate to what extent the following statements generally apply to you.	7-point scale from “Does not apply to me at all” (1) to “Fully applies to me” (7)
HC.1	Health is very important to me	
HC.2	I care a lot about health	
HC.3	I appreciate healthy food very much	
**Consumption frequency of pasta**	Please indicate your consumption frequency:	7-point scale from “Never” (1) to “Everyday” (7)
	- Regular	
	- Wholegrain	
**Pasta attribute**	Please indicate the importance of each of the following attributes when you choose pasta at the dining halls:	7-point scale from “Not at all important” (1) to “Extremely important” (7)
	- Type of cut	
	- Wholegrain attribute	
	- Type of toppings	
	- Healthiness	
**Perceptions of whole grain pasta**	In your opinion, a whole grain pasta is …	7-point semantic differential scale
P.1	Not tasty- Tasty	
P.2	Cheap-Expensive	
P.3	Not easily available-Easily available in the store I usually shop	
P.4	Not filling-Filling	
P.5	Not healthy-Healthy	
P.6	Not easily available-Easily available in the dining hall I usually eat	
**Attitude toward including whole grain pasta in the diet**	Including whole grain pasta in my diet over the next month will be:	7-point semantic differential scale
Att.1	Harmful-Beneficial	
Att.2	Foolish-Wise	
Att.3	Unnecessary-Essential	
Att.4	Difficult-Easy	
Att.5	Not tasty-Tasty	
**Willingness to include wholegrain pasta**	How likely would you be to willingly start including whole grain pasta in your diet over the next month?	7-point scale from “Extremely unlikely” (1) to “Extremely likely” (7)
**Self-perception of overall health**	How healthy do you consider yourself?	7-point scale from “Very bad” (1) to “Very well” (7)

### Appendix A.1. Factor Analysis of Self-Reported Healthy Eating, Interest in Healthy Eating, and Health Concern

Principal component analysis (PCA) with varimax rotation were performed on 3 consumers’ perceptions regarding healthy eating: self-reported healthy eating (HE), interest in healthy eating (IHE) and health concern (HC). Prior to performing PCA, the suitability of data for factor analysis was assessed. The Kaiser–Meyer–Olkin (KMO) statistics [64] and the statistical significance of the Bartlett’s test of sphericity [65] were used as criteria. Then, Cronbach’s alpha was used to check the reliability of the factors. Factor analysis results were shown in Table A2.

For HE, the first result of PCA indicated 2 factors with KMO 0.78 and the Bartlett’s test significant. The first factor consisted of HE.1, HE.3, HE.4, HE.5, HE.6 and HE.7 (Cronbach’s alpha = 0.74) and the second factor consisted of HE.2 only. Therefore, item HE.2 (“I eat bread, grains, pasta, rice or potatoes several times per day”) were taken off and the PCA analysis was performed with items HE.1, HE.3, HE.4, HE.5, HE.6 and HE.7 (KMO 0.78 with Bartlett’s test significant). The final result indicated one factor, so called “Healthy eating”, and its total variance explained was 43.9%. Originally, the standardized scores of item HE.2 was included in the cluster analysis, but it was not significant. Therefore, item HE.2 was not included in the final cluster analysis.

For IHE, prior to analyzing PCA, the scores of item IHE.3 (“I eat what I like and I do not worry about the healthiness of food”) were reversed. The first result of PCA indicated 1 factor with KMO 0.57, Bartlett’s test significant and Cronbach’s alpha = 0.69. However, the results of Cronbach’s alpha also suggested that the item IHE.3 (“I eat what I like and I do not worry about the healthiness of food”) should be taken off to improve reliability of the factor. Therefore, the PCA were performed with items IHE.1 and IHE.2 (KMO 0.50 with Bartlett’s test significant). The final result indicated one factor so called “Interest in Healthy Eating” and its total variance explained was 81.3%. While the reversed scores of item IHE.3 were standardized and later were used in cluster analysis.

For HC, the result of PCA indicated one factor with KMO 0.67 with Bartlett’s test significant. The factor was call “Health concern” and its total variance explained was 79.1%.

**Table A2:** Factor analysis results of healthy eating, interest in healthy eating, and health concern (N = 499).

	Perception	Median	Mean	SD	Factor Loading	Cronbach’s Alpha
**Healthy Eating**						**0.74**
HE.1	I eat a variety of foods originating mainly from plants, rather than animals	4	4.3	1.76	0.62	
HE.3	I eat a variety of vegetables and fruits, at least 0.9 lb (400 g) per day or 5 portions per day	4	4.2	1.62	0.72	
HE.4	I control my fat intake and replace most saturated fats with unsaturated vegetable oils or soft margarines	3	3.5	1.61	0.72	
HE.5	I replace fatty meat and meat products with beans, legumes, lentils, fish, poultry or lean meat	3	3.6	1.92	0.73	
HE.6	I select foods that are low in sugar, limiting the frequency of intake of sugary drinks and sweets	4	4.2	1.84	0.62	
HE.7	I control my salt intake and limit adding salt to my meals	4	3.7	1.80	0.55	
**Interest in Healthy Eating**						0.77
IHE.1	I always follow a healthy and balanced diet	4	4.0	1.50	0.90	
IHE.2	I am very particular about the healthiness of food	4	3.8	1.54	0.90	
**Health Concern**						**0.86**
HC.1	Health is very important to me	6	5.5	1.35	0.92	
HC.2	I care a lot about health	5	5.4	1.42	0.94	
HC.3	I appreciate healthy food very much	5	5.0	1.58	0.80	

Note: Participants were asked to indicate their opinion on the statements based on a 7-semantic scale (1 = does not apply to me at all, 7 = fully apply to me). Item HE.2 (“I eat bread, grains, pasta, rice or potatoes several times per day”) were taken off before performing final factor analysis.

### Appendix A.2. Factor Analysis of Attitude of Including Wholegrain Pasta in Diet and Perception of Wholegrain Pasta

For attitude of including wholegrain pasta in diet, the result of PCA indicated two factors with KMO 0.67 and the Bartlett’s test significant. Factor 1 (Cognitive attitude) consisted of Att.1, Att.2 and Att.3 and its total variance explained was 52.4%. Factor 2 (Affective attitude) consisted of Att.4 and Att.5 and its total variance explained was 21.8%. The total variance explained of 2 factors was 74.2%.

For perception of wholegrain pasta, the first result of PCA indicated two factors with KMO 0.71 and Bartlett’s test significant. Factor 1 consisted of P.1, P.3, P.4, P.5 and P.6 (Cronbach’s alpha = 0.709) and the second factor consisted of P.2 only. Therefore, item P.2 (“Cheap-Expensive) were taken off and the PCA analysis was performed with items P.1, P.3, P.4, P.5 and P.6 (KMO 0.71 with Bartlett’s test significant). The final result indicated one factor so called “Perception of whole grain pasta” and its total variance explained was 48.2%. Originally, the standardized scores of item P.2 was included in the logistic regression analysis but it was not significant. Therefore, item P.2 was not included in the final logistic regression analysis.

**Table A3:** Factor analysis results of attitude of including wholegrain pasta in the diet over the next month and perception of wholegrain pasta.

Attitude/Perception	Median	Mean	SD	Factor Loading	Cronbach’s Alpha
**Cognitive attitude ^1^**					**0.795**
Att.1 Harmful/Beneficial	5	5.3	1.37	0.89	
Att.2 Foolish/Wise	5	5.1	1.41	0.90	
Att.3 Unnecessary/Essential	4	3.9	1.59	0.70	
**Affective attitude ^1^**					**0.700**
Att.4 Difficult/Easy	5	5.0	1.67	0.86	
Att.5 Not tasty/Tasty	4	4.5	1.72	0.86	
**Perception of whole grain pasta ^2^**					**0.709**
P.1 Not tasty/Tasty	5	4.5	1.80	0.56	
P.3 Not easily available/Easily available in the store I usually shop	5	4.8	1.56	0.73	
P.4 Not filling/Filling	5	5.2	1.46	0.85	
P.5 Not healthy/Healthy	5	5.3	1.37	0.70	
P.6 Not easily available-Easily available in the dining hall I usually eat	5	4.7	1.73	0.59	

Note: ^1^ Participants were asked to indicate their attitude toward including whole grain pasta in the diet over the next month based on a 7-semantic scale. ^2^ Participants were asked to indicate their opinion on whole grain pasta based on a 7-semantic scale. Item P.2 (“Cheap/Expensive”) were taken off before performing final factor analysis.
